# Identification of Bioactive Natural Product from the Stems and Stem Barks of *Cornus walteri*: Benzyl Salicylate Shows Potential Anti-Inflammatory Activity in Lipopolysaccharide-Stimulated RAW 264.7 Macrophages

**DOI:** 10.3390/pharmaceutics13040443

**Published:** 2021-03-25

**Authors:** Dahae Lee, Akida Alishir, Tae Su Jang, Ki Hyun Kim

**Affiliations:** 1 College of Korean Medicine, Gachon University, Seongnam 13120, Korea; pjsldh@gachon.ac.kr; 2 School of Pharmacy, Sungkyunkwan University, Suwon 16419, Korea; akida.alishir@gmail.com; 3 College of Medicine, Dankook University, Cheonan 31116, Korea

**Keywords:** *Cornus walteri*, cornaceae, inflammation, nitric oxide, nuclear factor kappa B, inducible nitric oxide synthase, cyclooxygenase-2

## Abstract

*Cornus walteri* (Cornaceae), known as Walter’s dogwood, has been used to treat dermatologic inflammation and diarrheal disease in traditional oriental medicine. As part of an ongoing research project to discover natural products with biological activities, the anti-inflammatory potential of compounds from *C. walteri* in lipopolysaccharide (LPS)-stimulated mouse RAW 264.7 macrophages were explored. Phytochemical analysis of the methanol extract of the stem and stem bark of *C. walteri* led to the isolation of 15 chemical constituents. These compounds were evaluated for their inhibitory effects on the production of the proinflammatory mediator nitric oxide (NO) in LPS-stimulated macrophages, as measured by NO assays. The molecular mechanisms underlying the anti-inflammatory activity were investigated using western blotting. Our results demonstrated that among 15 chemical constituents, lupeol and benzyl salicylate inhibited NO production in LPS-activated RAW 264.7 macrophages. Benzyl salicylate was more efficient than *N*^G^-monomethyl-L-arginine mono-acetate salt (L-NMMA) in terms of its inhibitory effect. In addition, the mechanism of action of benzyl salicylate consisted of the inhibition of phosphorylation of IκB kinase alpha (IKKα), IκB kinase beta (IKKβ), inhibitor of kappa B alpha (IκBα), and nuclear factor kappa B (NF-κB) in LPS-stimulated macrophages. Furthermore, benzyl salicylate inhibited the expression of inducible nitric oxide synthase (iNOS) and cyclooxygenase-2 (COX-2). Taken together, these results suggest that benzyl salicylate present in the stem and stem bark of *C. walteri* has potential anti-inflammatory activity, supporting the potential application of this compound in the treatment of inflammatory diseases.

## 1. Introduction

Inflammatory responses are the biological reactions of body tissues to stimulation by various cytokines secreted by a range of other cells [1]. Macrophages are inflammatory cells that play an important role in the promotion of inflammatory responses by producing nitric oxide (NO) as a proinflammatory mediator [2]. Therefore, the quantities of inflammatory mediators and the activity levels in the signaling pathways that regulate these mediators in macrophages may provide evidence of the effects of anti-inflammatory agents.

As part of an ongoing research project to discover bioactive compounds in diverse natural resources [3,4,5,6,7,8,9], we investigated candidate phytochemicals from a methanol extract of the stem and stem bark of *Cornus walteri* to explore their anti-inflammatory potential by using a mouse macrophage cell line (RAW 264.7). *Cornus walteri* Wanger, belonging to the family Cornaceae, is known as Walter’s dogwood. This plant is a deciduous shrub grown in eastern Asia, especially in China and Korea, as an economic crop for high-grade furniture and agricultural tools. Traditionally, its fruits and leaves have been used in folk medicine to treat the dermatologic inflammation caused by lacquer poisoning, as indicated in the Chinese Materia Medica [10]. Its leaves have been also used as a Korean medicine herb to treat diarrhea [11]. Previous pharmacological studies of this plant have reported that *C. walteri* extracts exhibit therapeutic properties, including anti-hyperglycemic and anti-obesity effects and anti-inflammatory and antioxidant properties [12,13]. A recent study revealed that such extracts protect reconstituted human skin against photoaging caused by ultraviolet B (UVB) [10]. In addition, previous chemical investigations of *C. walteri* have demonstrated the presence of diverse types of chemical constituent, including lignans and flavonoids [14,15]. However, few previous studies have been carried out to investigate the chemical constituents of *C. walteri*, despite many studies on the pharmacological effects of *C. walteri* extracts.

In this context, our group focused on the potentially bioactive constituents of *C. walteri*. In our previous chemical investigation of *C. walteri*, we identified triterpenoids and δ-valerolactones, which showed cytotoxicity against several human cancer cell lines, using bioassay-guided fractionation of the methanol extract of *C. walteri* [16,17,18,19]. In particular, we found that betulinic acid, a triterpenoid, reduced the viability of A2780 human ovarian carcinoma cells and induced apoptotic cell death through both extrinsic and intrinsic apoptosis pathway [19]. Furthermore, we identified new tirucallane triterpenoids (cornusalterins N-P) in *C. walteri*, along with bioactive tirucallane triterpenoids that control adipocyte and osteoblast differentiation [20]. A nephroprotective agent has also been identified in *C. walteri* extracts using a model of cisplatin-induced cell death in LLC-PK1 kidney proximal tubule cell line; it was found to decrease the proteins involved in intrinsic apoptosis pathway [21]. As *C. walteri* extract has been known to inhibit NO production in RAW 264.7 macrophages stimulated with lipopolysaccharide (LPS) [22], the present study was carried out for further investigation of the methanol extracts of the stem and stem bark of *C. walteri* to identify potential anti-inflammatory constituents. Herein, we describe the isolation and structural characterization of compounds **1**–**15** and evaluate their anti-inflammatory activity in LPS-stimulated RAW 264.7 macrophages.

## 2. Materials and Methods

### 2.1. Plant Material, Extraction and Isolation of Compounds **1–15**

The information for plant material and extraction of the plant is included in Supplementary Materials. The detailed procedure for the isolation of compounds **1–15** is also included in Supplementary Materials.

### 2.2. RAW 264.7 Cells Culture

A mouse macrophage cell line, RAW 264.7 (American Type Culture Collection, Rockville, MD, USA), was cultured in DMEM (Manassas, VA, USA) containing 4 mM L-glutamine, antibiotics (1% penicillin/streptomycin), and 10% fetal bovine serum in humidified air environment at 37 °C in a 5% CO_2_.

### 2.3. Measurement of Viability of RAW 264.7 Cells

RAW 264.7 cells (3 × 10^4^ cells/well) were exposed to the indicated concentrations of compounds **1**–**15** for 24 h at 37 °C and incubated for an additional 40 min with Ez-Cytox solution (Daeil Lab Service Co., Seoul, Korea). Optical density at 450 nm was determined using a spectrophotometer microplate (PowerWave XS; Bio-Tek Instruments, Winooski, VT, USA).

### 2.4. Measurement of NO Produced by RAW 264.7 Cells

RAW 264.7 cells (3 × 10^4^ cells/well) were exposed to the indicated concentrations of compounds **1**–**15** for 1 h and then incubated for an additional 24 h with LPS (1 μg/mL). At the end of the incubation, each culture supernatant was blended with the Griess reagent to determine NO production by RAW 264.7 cells. Optical density at 540 nm of mixture was determined using a spectrophotometer microplate (PowerWave XS; Bio-Tek Instruments, Winooski, VT, USA).

### 2.5. Western Blot Analysis

RAW 264.7 cells (4 × 10^5^ cells/well) were exposed to the indicated concentrations of benzyl salicylate (**15**) for 1 h and then incubated for an additional 24 h with LPS (1 μg/mL). At the end of the incubation, the RAW 264.7 cells were lysed with lysis buffer (Cell Signaling Technology, Beverly, MA, USA), supplemented with 1 mM phenylmethylsulfonyl fluoride, for 20 min. For western blot analysis, 20 μg of the total protein from the cell lysate was separated by 10% sodium dodecyl sulfate–polyacrylamide gel electrophoresis (SDS-PAGE). The proteins were electro-transferred to a polyvinylidene fluoride (PVDF) membrane. Each PVDF membrane was probed with primary antibodies (Cell Signaling Technology, Beverly, MA, USA) overnight, incubated with horse radish peroxidase-conjugated anti-rabbit antibodies (Cell Signaling, Beverly, MA, USA) for 1 h at room temperature, and visualized using an enhanced chemiluminescence detection reagent (GE Healthcare, Little Chalfont, UK). Western blot signals were detected by FUSION Solo Chemiluminescence System (PEQLAB Biotechnologie GmbH, Erlangen, Germany).

### 2.6. Statistical Analysis

All assays were performed in triplicate and repeated at least three times. All data are presented as the mean ± standard deviation (SD). Statistical significance was determined using one-way analysis of variance (ANOVA) and multiple comparisons with the Bonferroni correction. A *p* value of < 0.05 indicated statistical significance. All analyses were performed using SPSS Statistics ver. 19.0 (SPSS Inc., Chicago, IL, USA).

## 3. Results

### 3.1. Isolation and Identification of the Compounds

**Figure 1:**
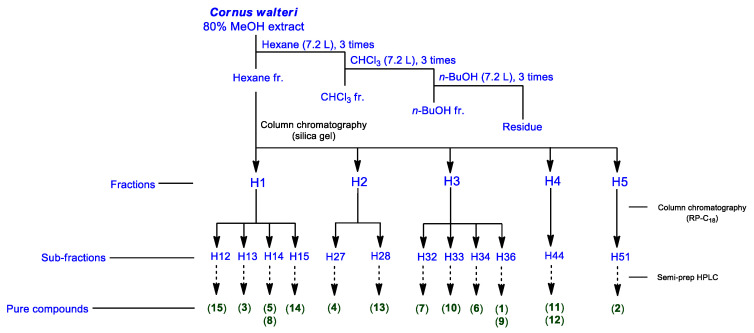
Separation scheme of compounds **1**–**15**. 5α-Stigmast-3,6-dione (**1**), 3β-sitostanol (**2**), 6α-hydroxy-β-sitostenone (**3**), 6β-hydroxysitostenone (**4**), norphytan (**5**), phytone (**6**), methyl 3-*O*-acetylbetulinate (**7**), 3-*O*-acetylbetulin (**8**), sitostenone (**9**), leucophyllone (**10**), lupeol (**11**), lupenone (**12**), betulinic acid (**13**), betulinic acid methyl ester (**14**), and benzyl salicylate (**15**). MeOH, methanol; BuOH, butanol; RP, reversed phase; HPLC, high-performance liquid chromatography.

**Figure 2:**
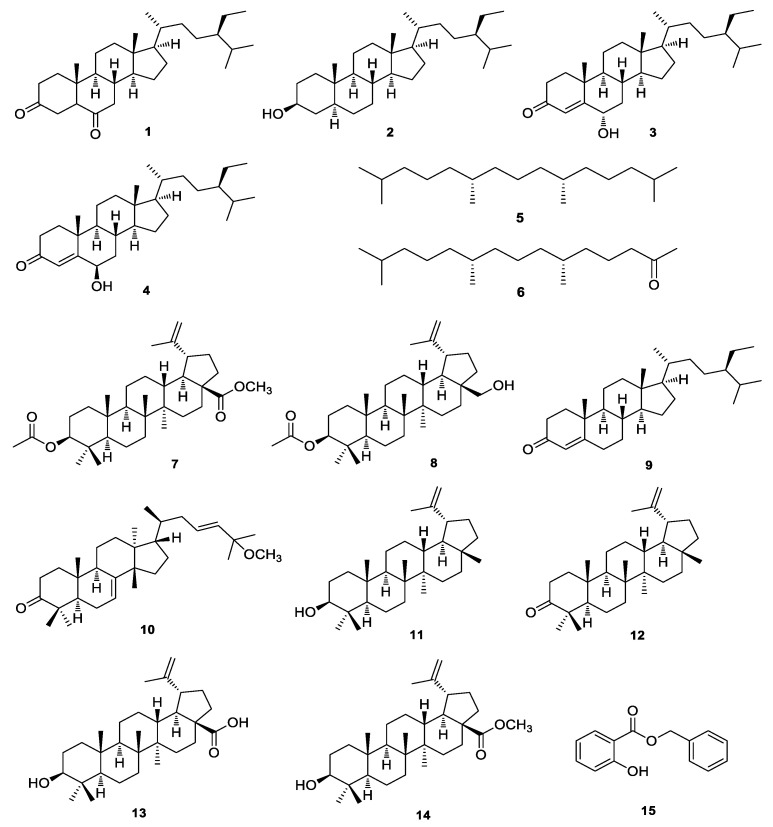
Chemical structures of compounds **1**–**15**. 5α-Stigmast-3,6-dione (**1**), 3β-sitostanol (**2**), 6α-hydroxy-β-sitostenone (**3**), 6β-hydroxysitostenone (**4**), norphytan (**5**), phytone (**6**), methyl 3-*O*-acetylbetulinate (**7**), 3-*O*-acetylbetulin (**8**), sitostenone (**9**), leucophyllone (**10**), lupeol (**11**), lupenone (**12**), betulinic acid (**13**), betulinic acid methyl ester (**14**), and benzyl salicylate (**15**).

The stem and stem bark of *C. walteri* were extracted with 80% methanol under reflux to obtain the methanol extract by rotary evaporation. The methanol extract was sequentially partitioned using the organic solvents hexane, CHCl_3_, and *n*-butanol to yield each solvent fraction (Figure 1). TLC analysis of the solvent fractions determined that the hexane-soluble fraction possesses the major spots. Phytochemical analysis of the hexane fraction was carried out by applying column chromatography and high-performance liquid chromatography (HPLC) as well as LC/MS analysis. Semi-preparative HPLC separation yielded 15 compounds (Figure 1): 5α-stigmast-3,6-dione (**1**) [23], 3β-sitostanol (**2**) [24], 6α-hydroxy-β-sitostenone (**3**) [25], 6β-hydroxysitostenone (**4**) [26], norphytan (**5**) [27], phytone (**6**) [28], methyl 3-*O*-acetylbetulinate (**7**) [29], 3-*O*-acetylbetulin (**8**) [30], sitostenone (**9**) [31], leucophyllone (**10**) [16], lupeol (**11**) [32], lupenone (**12**) [33], betulinic acid (**13**) [34], betulinic acid methyl ester (**14**) [35], and benzyl salicylate (**15**) [36]. The structures of compounds **1–15** (Figure 2) were determined by comparing their ^1^H and ^13^C NMR spectra with those previously reported in the literature [16,23,24,25,26,27,28,29,30,31,32,33,34,35,36], and by LC/MS analysis.

### 3.2. Effects of Compounds **1–15** on Nitric Oxide (NO) Production

**Figure 3:**
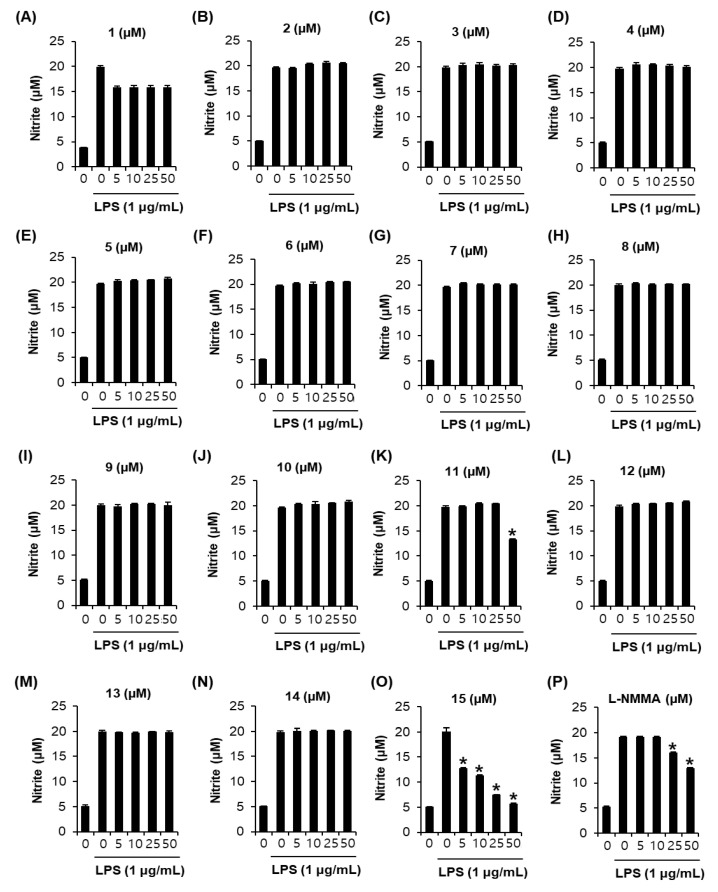
Effects of compounds **1–15** and *N*^G^-monomethyl-L-arginine mono-acetate salt (L-NMMA) in RAW 264.7 mouse macrophages treated with lipopolysaccharide (LPS). (**A**–**P**) The effects of compounds **1–15** and L-NMMA in RAW 264.7 mouse macrophages treated with LPS were investigated (mean ± SD, *p < 0.05 compared to group treated with 1 μg/mL LPS alone). 5α-Stigmast-3,6-dione (**1**), 3β-sitostanol (**2**), 6α-hydroxy-β-sitostenone (**3**), 6β-hydroxysitostenone (**4**), norphytan (**5**), phytone (**6**), methyl 3-*O*-acetylbetulinate (**7**), 3-*O*-acetylbetulin (**8**), sitostenone (**9**), leucophyllone (**10**), lupeol (**11**), lupenone (**12**), betulinic acid (**13**), betulinic acid methyl ester (**14**), and benzyl salicylate (**15**).

The inhibitory effects of compounds **1–15** on NO production in LPS-activated RAW 264.7 macrophages were investigated. Among these compounds, only lupeol (**11**) and benzyl salicylate (**15**) attenuated nitrite concentration in LPS-activated RAW 264.7 cells (Figure 3). As shown in Figure 3K, compared with the LPS-only treatment group (19.81 ± 0.22 μM), coincubation with 50 μM lupeol (**11**) and LPS resulted in a 13.26 ± 0.05 μM lower nitrite concentration. As shown in Figure 3O, compared with the LPS-only treatment group (20.13 ± 0.66 μM), coincubation with 50 μM benzyl salicylate (**15**) and LPS produced a 5.74 ± 0.09 μM lower nitrite concentration with an IC_50_ value of 5.51 ± 0.39 μM. As shown in Figure 3P, after coincubation with 50 μM of the *N*^G^-monomethyl-L-arginine mono-acetate salt (L-NMMA) and LPS, the nitrite concentration was found to be 12.91 ± 0.14 μM lower than that in the LPS-only treatment group (19.16 ± 0.07 μM).

### 3.3. Effects of Benzyl Salicylate (**15**) on the LPS-Induced Expression of IKKα/β, I-κBα, and NF-κB in RAW 264.7 Mouse Macrophages

**Figure 4:**
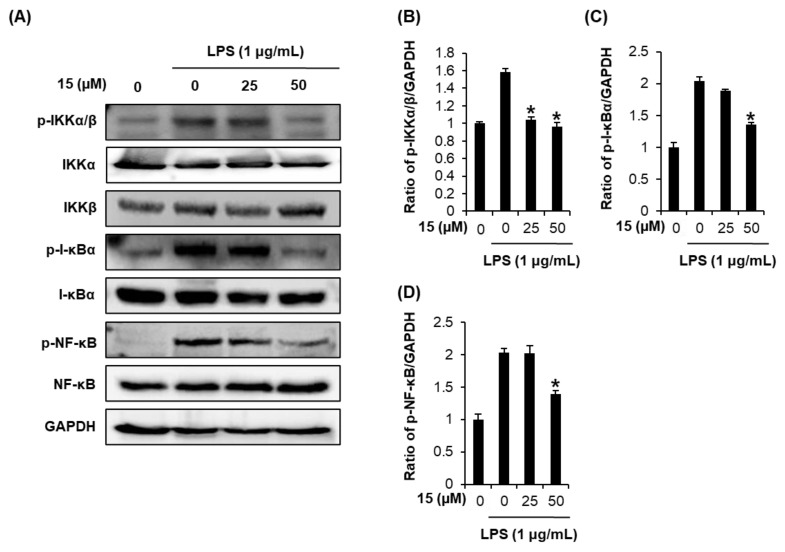
Effects of benzyl salicylate (**15**) on the expression of IκB kinase alpha and beta (IKKα/β), inhibitor of kappa B alpha (I-κBα), and nuclear factor kappa B (NF-κB) in RAW 264.7 mouse macrophages treated with lipopolysaccharide (LPS). (**A**) Representative western blots showing protein expressions of IKKα/β, phospho-IKKα/β (p-IKKα/β), I-κBα, phospho-IKKα/β (p-I-κBα), NF-κB, and glyceraldehyde-3-phosphate dehydrogenase (GAPDH). (**B**–**D**) Quantitative bar chart for each protein’s expression level (mean ± SD, *p < 0.05 compared to group treated with 1 μg/mL LPS alone).

The effect of benzyl salicylate (**15**) on LPS-induced expression of IκB kinase alpha and beta (IKKα/β), inhibitor of kappa B alpha (I-κBα), and nuclear factor kappa B (NF-κB) were analyzed by Western blot. We found that the LPS-stimulated RAW264.7 cells overexpressed IKKα/β, I-κBα, and NF-κB, whereas co-incubation with benzyl salicylate (**15**) inhibited this overexpression (Figure 4).

### 3.4. Effects of Benzyl Salicylate (**15**) on the LPS-Induced Expression of iNOS and COX-2 in RAW 264.7 Mouse Macrophages

**Figure 5:**
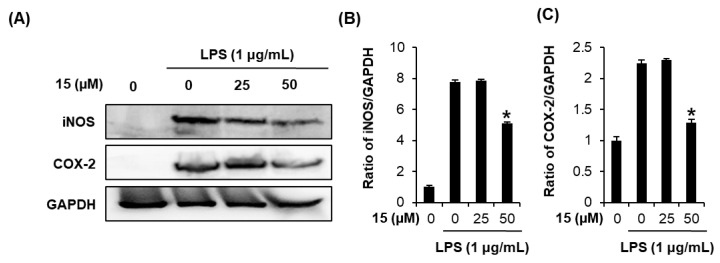
Effects of benzyl salicylate (**15**) on the expression of inducible nitric oxide synthase (iNOS) and cyclooxygenase-2 (COX-2) in RAW 264.7 mouse macrophages treated with lipopolysaccharide (LPS). (**A**) Representative western blots showing protein expressions of iNOS, COX-2, and glyceraldehyde-3-phosphate dehydrogenase (GAPDH). (**B**–**C**) Quantitative bar chart for each protein’s expression level (mean ± SD, * *p* < 0.05 compared to group treated with 1 μg/mL LPS alone).

In a subsequent experiment, we found that LPS-stimulated RAW264.7 cells overexpressed iNOS and COX-2, whereas coincubation with benzyl salicylate (**15**) inhibited this overexpression (Figure 5).

## 4. Discussion

In previous study, *C. walteri* extract has been known to inhibit nitric oxide (NO) production in RAW 264.7 macrophages stimulated with lipopolysaccharide (LPS) [22]. However, little is known about the corresponding bioactive compounds isolated from *C. walteri* and their possible mechanism of action. Phytochemical analysis in the present study was conducted to isolate the 15 chemical constituents from hexane fraction of the stem and stem bark of *C. walteri*.

Nitric oxide (NO) is synthesized during the inflammatory response to lipopolysaccharide (LPS), an endotoxin; NO production has been extensively used as a model for the study of inflammation in the mouse macrophage cell line RAW 264.7 [37,38,39,40]. Overproduction of NO under abnormal conditions causes an inflammatory response [37,38,39,40,41,42]. In the present study, the inhibitory effects of compounds 1–15 on NO production in LPS-activated RAW 264.7 macrophages were investigated [43]. Among these compounds, only lupeol (**11**) and benzyl salicylate (**15**) attenuated nitrite concentration in LPS-activated RAW 264.7 cells. Interestingly, benzyl salicylate (**15**) was more efficient than L-NMMA in inhibiting NO production in LPS-activated RAW 264.7 macrophages. In previous studies, benzyl salicylate was shown to be an active compound with estrogenic activity [44], and a protective agent against cisplatin-induced damage to cells of the LLC-PK1 kidney proximal tubule cell line [21]. However, to the best of our knowledge, there are no reports available regarding the inhibition of NO production in LPS-activated RAW 264.7 macrophages by benzyl salicylate. To investigate the mechanism that inhibits NO production by benzyl salicylate in LPS-activated RAW 264.7 macrophages, western blot analysis was performed.

Upon stimulation by LPS or proinflammatory cytokines, the two catalytic subunits of IKK (IKKα and IKKβ) contribute to the phosphorylation of I-κBα, which enables the activation of NF-κB [45]. NF-κB is a key regulator of the expression of inflammatory cytokines, such as iNOS and COX-2 [46]. In the present study, the LPS-stimulated RAW264.7 cells overexpressed IKKα/β, I-κBα, and NF-κB, whereas co-incubation with benzyl salicylate (**15**) inhibited this overexpression. Thus, benzyl salicylate (**15**) inhibited LPS-induced NO production through the inhibition of NF-κB/I-κBα pathway in RAW 264.7 cells.

**Figure 6:**
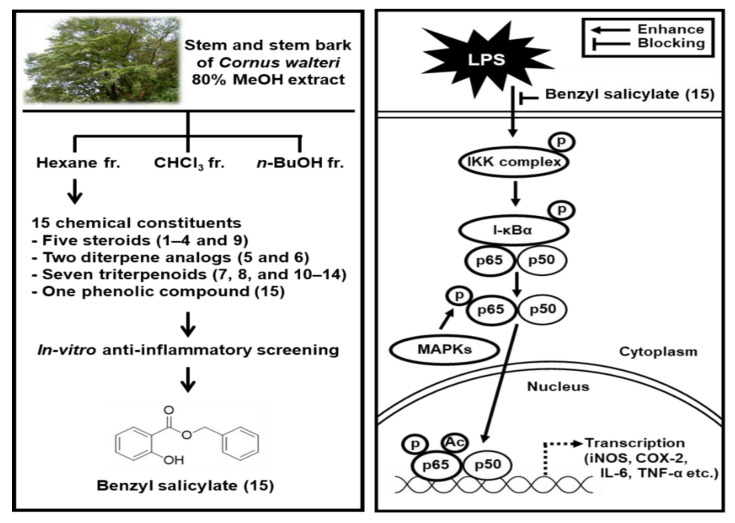
Schematic pathway for the potential role of benzyl salicylate (**15**) isolated from *C. walteri* in inflammatory responses. LPS, lipopolysaccharide; p, phosphorylated; IKK, IκB kinase alpha; IκBα, inhibitor of kappa B alpha; p56 and p50, cellular proteins; MAPK, mitogen-activated protein kinase; Ac, activated; TNF-α, tumor necrosis factor alpha; IL-6, interleukin 6; COX-2, cyclooxygenase-2; iNOS, inducible nitric oxide synthase.

Activation of NF-κB increases iNOS expression by increasing its binding to the iNOS promoter to produce NO [47,48]. This simultaneously activates COX-2 synthesis [49]. In the present study, LPS-stimulated RAW264.7 cells overexpressed iNOS and COX-2, whereas coincubation with benzyl salicylate (**15**) inhibited this overexpression. These findings suggest that benzyl salicylate (**15**) inhibited LPS-induced NO production by reducing iNOS and COX-2 expression in LPS-stimulated RAW 264.7 cells. Furthermore, they suggest that benzyl salicylate (**15**) simultaneously inhibited LPS-induced inflammatory responses by blocking the degradation of I-κBα and thereby inhibiting NF-κB, based on the experimental evidence associated with the inhibition of COX-2 and iNOS expression (Figure 6). Although its bioavailability and bio-accessibility should be verified through additional studies, including animal experiments, our data suggest that benzyl salicylate (**15**) has potential as an anti-inflammatory agent for the treatment of inflammatory diseases.

## 5. Conclusions

In this study, we identified fifteen compounds (**1–15**) present in the stem and stem bark of *C. walteri*. We found that benzyl salicylate (**15**) inhibited NO production in LPS-stimulated RAW 264.7 macrophages, mediated by inhibition of the expression of the proteins IKKα/β, I-κBα, NF-κB, iNOS, and COX-2, which eventually produce NO. Based on these findings, we conclude that benzyl salicylate (**15**) possesses potential anti-inflammatory effects, supporting the application of this compound in the treatment of inflammatory diseases.

## Supplementary Materials

The following are available online at https://www.mdpi.com/article/10.3390/pharmaceutics13040443/s1, General experimental procedure, plant material, and extraction and isolation procedures.
