# Supplementary material for: Identification of Bioactive Natural Product from the Stems and Stem Barks of Cornus walteri: Benzyl Salicylate Shows Potential Anti-Inflammatory Activity in Lipopolysaccharide-Stimulated RAW 264.7 Macrophages
Source: Pharmaceutics. 2021 Mar 25;13(4):443. doi: 10.3390/pharmaceutics13040443 (PMC8064495; doi:10.3390/pharmaceutics13040443)
Supplement: Supplementary file 1 [file pharmaceutics-13-00443-s001.pdf]

pharmaceutics

MDPI

Supplementary Materials: Identification of Bioactive Natural Product from the Stems and Stem Barks of *Cornus walteri*: Benzyl Salicylate Shows Potential Anti-inflammatory Activity in Lipopolysaccharide-stimulated RAW 264.7 Macrophages

Dahae Lee [1,2,†], Akida Alishir [2,†], Tae Su Jang [3,*], and Ki Hyun Kim [2,*]

General Experimental Procedure

A Jasco P-1020 polarimeter was used to measure optical rotation. IR spectra were obtained using a Bruker IFS-66/S FT-IR spectrometer. UV spectra were obtained using an Agilent 8453 UV–visible spectrophotometer. Nuclear magnetic resonance (NMR) spectra were recorded with a Varian UNITY INOVA 500 NMR spectrometer operating at 500 MHz (^{1}H) and 125 MHz (^{13}C), with chemical shifts given in ppm (δ) for ^{1}H and ^{13}C NMR analyses. Semi-preparative high-performance liquid chromatography (HPLC) was performed using a Gilson 306 pump with a Shodex refractive index detector and a Phenomenex Luna Phenyl-Hexyl column (250 × 10 mm, 10 μm; flow rate: 2 mL/min; Phenomenex, Torrance, CA, USA). LC/MS analysis was carried out using an Agilent 1200 Series HPLC system equipped with a diode array detector and a 6130 Series ESI mass spectrometer with an analytical Kinetex C_{18} 100 Å column (100 × 2.1 mm, 5 μm; flow rate: 0.3 mL/min; Phenomenex). Column chromatography was conducted on a silica gel 60 column (Merck, 230–400 mesh) and reversed-phase (RP)-C_{18} silica gel column (Merck, 230–400 mesh). Molecular sieve column chromatography was performed on a Sephadex LH-20 column (Pharmacia, Uppsala, Sweden). The packing material used for open-column chromatography was Diaion HP-20 (Mitsubishi Chemical, Tokyo, Japan). Merck precoated silica gel F_{254} plates and RP-C_{18} F_{254s} plates were used for thin-layer chromatography (TLC). The chromatographic spots were visualized under UV light (254 and 365 nm) or by heating after spraying with anisaldehyde sulfuric acid.

Plant Material

Stem and stem bark of *C. walteri* were collected from Jeju Island, Korea, in October 2005, and identified by one of the authors (K. H. Kim). A voucher specimen (SKKU 2005-10a) was deposited at the herbarium of the School of Pharmacy, Sungkyunkwan University, Suwon, Korea.

Extraction and Isolation

Dried and chopped *C. walteri* sample (2.5 kg) were extracted in 80% aqueous methanol (2 × 6 h, under reflux) and filtered. The filtrate was concentrated under reduced pressure by using a rotavapor to obtain a methanol extract (220 g), which was suspended in distilled H_2O (7.2 L). The extract was successively solvent-partitioned with hexane, $CHCl_3$, and *n*-butanol. Three fractions with increasing polarity were obtained: hexane-soluble (9.5 g), $CHCl_3$-soluble (25.0 g), and *n*-butanol-soluble (43.0 g). The residue (9.5 g) from the hexane fraction was subjected to a silica gel (230–400 mesh, 300 g) column in a gradient solvent system of hexane-ethyl acetate (3:1 to 1:1) to yield five fractions (H1, H2, H3, H4, and H5). Fraction H1 (4.0 g) was chromatographed using an RP-C18 silica gel column with 100% methanol to obtain five fractions (H11–H15). Fraction H12 (800 mg) was separated on a Sephadex LH-20 column with a solvent system of CH_2Cl_2-methanol (1:1) to produce two subfractions, H121 and H122. Fraction H122 (50 mg) was purified using silica gel Waters Sep-Pak Vac 6 cc with hexane-ethyl acetate (30:1) to obtain compound **15** (12 mg).

Fraction H13 (800 mg) was subjected to silica gel column chromatography using a gradient solvent system of hexane-ethyl acetate (7:1 to 1:1, v/v) to obtain six subfractions (H131–H136). Subfraction H131 (100 mg) was separated by semi-preparative normal-phase HPLC (Apollo Silica column, 250 × 10.0 mm, 5 μm, flow rate: 2 mL/min) by using an isocratic elution of hexane-ethyl acetate (18:1, v/v) to yield compound **3** (6 mg). Fraction H14 (300 mg) was subjected to silica gel column chromatography and eluted with a gradient solvent system of hexane-ethyl acetate (16:1 to 1:1, v/v), and five subfractions (H141–H145) were acquired. Compound **8** (40 mg) was obtained from purified subfraction H142 (50 mg) via semi-preparative normal-phase HPLC (Apollo Silica column, 250 × 10.0 mm, 5 μm, flow rate: 2 mL/min) with an isocratic solvent system of hexane-ethyl acetate (28:1, v/v). Subfraction H144 (50 mg) was isolated using semi-preparative normal-phase HPLC (Apollo Silica column, 250 × 10.0 mm, 5 μm, flow rate: 2 mL/min) with an isocratic solvent system of hexane-ethyl acetate (12:1, v/v) to yield compound **5** (30 mg). Fraction H15 (100 mg) was passed into a C18 Sep-Pak column eluted with an isocratic solvent system of hexane-ethyl acetate (10:1, v/v) and further purified using semi-preparative normal-phase HPLC (Apollo Silica column, 250 × 10.0 mm, 5 μm, flow rate: 2 mL/min) in an isocratic solvent system of hexane-ethyl acetate (20:1, v/v) to obtain compound **14** (7 mg). Eight fractions (H21–H28) were obtained by chromatography of fraction H2 (2.5 g) with an RP-C18 silica gel column in 100% methanol. Fraction H27 (150 mg) was isolated using semi-preparative normal-phase HPLC (Apollo Silica column, 250 × 10.0 mm, 5 μm, flow rate: 2 mL/min) and eluted with an isocratic solvent system of hexane-ethyl acetate (8:1, v/v) to obtain compound **4** (60 mg). Fraction H28 (100 mg) was purified by semi-preparative normal-phase HPLC (Apollo Silica column, 250 × 10.0 mm, 5 μm, flow rate: 2 mL/min) in an isocratic elution of hexane-ethyl acetate (8:1, v/v) to yield compound **13** (25 mg). Fraction H3 (1.7 g) was separated using an RP-C18 silica gel column and eluted with 100% methanol to obtain six fractions (H31–H36). Fraction H32 (200 mg) was applied to a reversed-phase Lobar column in an isocratic solvent system of hexane-ethyl acetate (4:1, v/v) to obtain four subfractions (H321–H324). Compound **7** (4 mg) was obtained from subfraction H321 (150 mg), which was purified using semi-preparative reversed-phase HPLC (Econosil C18 column, 250 × 10.0 mm, 5 μm, flow rate: 2 mL/min) with an isocratic elution (95% methanol). Fraction H33 (400 mg) was isolated using a reversed-phase Lobar column in an isocratic solvent system of hexane-ethyl acetate (4:1, v/v) to obtain five subfractions (H331–H335). Fraction H331 (50 mg) was passed over a semi-preparative reversed-phase HPLC (Econosil C18 column, 250 × 10.0 mm, 5 μm, flow rate: 2 mL/min) and eluted with an isocratic, 100% methanol solvent system to obtain compound **10** (7 mg). Compound **6** (30 mg) was isolated from fraction H34 (120 mg) by semi-preparative normal-phase HPLC (Apollo Silica column, 250 × 10.0 mm, 5 μm, flow rate: 2 mL/min) with isocratic elution using hexane-ethyl acetate (3:1, v/v). Fraction H36 (120 mg) was separated using semi-preparative normal-phase HPLC (Apollo Silica column, 250 × 10.0 mm, 5 μm, flow rate: 2 mL/min) in an isocratic solvent system of hexane-ethyl acetate (4:1, v/v) to obtain compounds **9** (5 mg) and **1** (8 mg). Fraction H4 (1.3 g) was applied to an RP-C18 silica gel column and eluted with 100% methanol to obtain six fractions (H41–H46). Compounds **11** (25 mg) and **12** (5 mg) were isolated from fraction H44 (120 mg) by semi-preparative normal-phase HPLC (Apollo Silica column, 250 × 10.0 mm, 5 μm, flow rate: 2 mL/min) in an isocratic solvent system of hexane-ethyl acetate (2:1, v/v). Fraction H5 (500 mg) was loaded onto an RP-C18 silica gel column and eluted with an isocratic solvent system of 85% methanol to obtain five fractions (H51–H55). Fraction H51 (150 mg) was further separated using a Sephadex-LH20 column, followed by elution with CHCl3-methanol (1:1, v/v), and then purification using semi-preparative reversed-phase HPLC (Econosil C18 column, 250 × 10.0 mm, 5 μm, flow rate: 2 mL/min) with an isocratic solvent system of 100% methanol, producing compound **2** (6 mg).
